# Polarization and wavelength routers based on diffractive neural network

**DOI:** 10.1007/s12200-024-00126-2

**Published:** 2024-07-16

**Authors:** Xiaohong Lin, Yulan Fu, Kuo Zhang, Xinping Zhang, Shuai Feng, Xiaoyong Hu

**Affiliations:** 1 School of Physics and Optoelectronic Engineering, Beijing University of Technology, Beijing, 100124 China; 2 School of Science, Minzu University of China, Beijing, 100081 China; 3 State Key Laboratory for Mesoscopic Physics and Department of Physics, Collaborative Innovation Center of Quantum Matter, Beijing Academy of Quantum Information Sciences, Nano-Optoelectronics Frontier Center of Ministry of Education, Peking University, Beijing, 100871 China; 4 Collaborative Innovation Center of Extreme Optics, Shanxi University, Taiyuan, 030006 China; 5 Peking University Yangtze Delta Institute of Optoelectronics, Nantong, 226010 China

**Keywords:** Optical diffractive neural network, All-optical routers, Polarization degree of freedom, Wavelength degree of freedom

## Abstract

In the field of information processing, all-optical routers are significant for achieving high-speed, high-capacity signal processing and transmission. In this study, we developed three types of structurally simple and flexible routers using the deep diffractive neural network (D^2^NN), capable of routing incident light based on wavelength and polarization. First, we implemented a polarization router for routing two orthogonally polarized light beams. The second type is the wavelength router that can route light with wavelengths of 1550, 1300, and 1100 nm, demonstrating outstanding performance with insertion loss as low as 0.013 dB and an extinction ratio of up to 18.96 dB, while also maintaining excellent polarization preservation. The final router is the polarization-wavelength composite router, capable of routing six types of input light formed by pairwise combinations of three wavelengths (1550, 1300, and 1100 nm) and two orthogonal linearly polarized lights, thereby enhancing the information processing capability of the device. These devices feature compact structures, maintaining high contrast while exhibiting low loss and passive characteristics, making them suitable for integration into future optical components. This study introduces new avenues and methodologies to enhance performance and broaden the applications of future optical information processing systems.

**Graphical Abstract:**

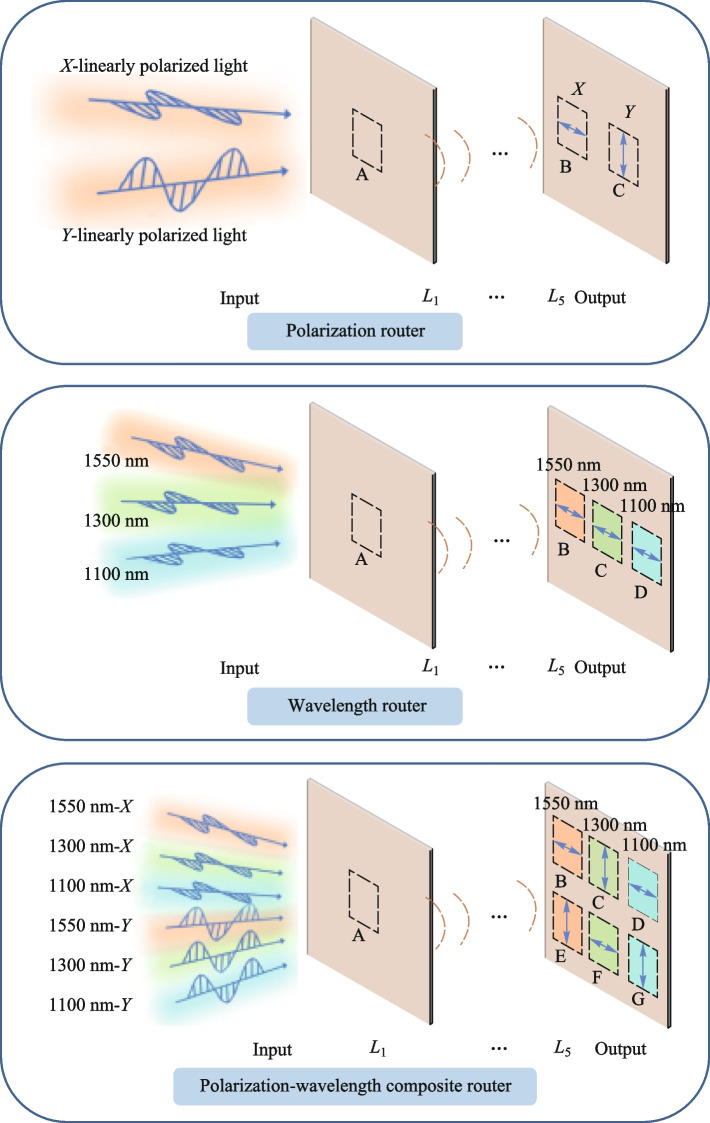

**Supplementary Information:**

The online version contains supplementary material available at 10.1007/s12200-024-00126-2.

## Introduction

In recent years, with the rapid development of high-traffic services such as the Internet of Things [1–4] and cloud computing [5–10], there has been a growing demand for efficient, high-capacity signal transmission and processing. It is foreseeable that the demand for information will continue to expand, posing formidable challenges to existing communication and computing technologies. Traditional metal-wire networks suffer from issues such as high power consumption, low bandwidth, and high latency, which constrain further advancements in the performance of multi-core processor systems. Therefore, to further develop high-performance central processing units for supercomputers, leveraging optical interconnection technology [11–18] is essential. Optical interconnection systems offer high interconnect density and low power consumption, serving as a potential solution for electronic devices requiring high data capacity. In all-optical communication systems, the strategy required for fully integrated optical networks is to establish complete links between different nodes in the network. However, the overall performance of the network is affected by the challenge of establishing complete connections and the complexity of data transmission between nodes. As a significant component in optical interconnections, optical routers [19, 20] play an indispensable role in facilitating efficient data transmission across optical networks. A multitude of methodologies have been proposed to realize the all-optical router. These encompass diverse approaches, ranging from nanoring resonators [21] to wavelength routers integrating wavelength converters and delay interferometers [22], and even orbital angular momentum routers achieved through the utilization of dual tripod atomic systems [23]. These optical designs are inherently modular, demanding the integration of diverse and various optical components to achieve different functionalities.

To realize the all-optical router, we propose an intuitive approach based on the deep diffractive neural network (D^2^NN). D^2^NN is a series of successive diffractive layers designed in a computer using error backpropagation and stochastic gradient descent methods [24]. Each diffractive layer consists of an array of passive pixels, with each pixel serving as a parameter learned by the computer. These parameters are used to adjust the phase and amplitude of the electric field. As light passes through the diffractive plate, variations in the transmission or reflection coefficients at different positions on the plate cause changes in the phase and amplitude of the light. D^2^NN operates solely on optical diffraction without the need for electrical power, thereby creating an efficient and fast approach for implementing machine learning tasks. For example, Ding et al. utilized D^2^NN to implement logical [25, 26] and trigonometric [27] operations. Their device featured a simple and compact structure with strong practical applicability. D^2^NN has been applied in image recognition [24, 28–44], optical logic operations [25, 26, 45–48], terahertz pulse shaping [49], phase retrieval [50], image reconstruction [51–53], and other fields.

In this paper, we present the structurally simple and flexible multi-degree-of-freedom routers achieved through the utilization of D^2^NN. D^2^NN integrates optical structural units into a compact and scalable system, effectively reducing overall complexity and enhancing the practicality and feasibility of implementing the realized all-optical routers. This study utilizes the D^2^NN model to integrate multiple degrees of freedom, realizing the polarization router, wavelength router, and polarization-wavelength composite router. The polarization router can route two orthogonally polarized light beams. The wavelength router can route light with wavelengths of 1550, 1300, and 1100 nm under fixed polarization conditions, with an insertion loss as low as 0.013 dB, an extinction ratio of up to 18.96 dB, and excellent polarization maintenance. The polarization-wavelength composite router can route six types of input light, which are formed by pairwise combinations of three wavelengths (1550, 1300, and 1100 nm) and two orthogonal linearly polarized lights. By integrating wavelength and polarization degrees of freedom, the information processing capability of the device is enhanced. These implemented routers are ultra-compact, maintaining high contrast while exhibiting low loss and passive characteristics, providing new ideas and methods for improving performance, and expanding the applications of future optical information processing systems.

## Methods

### Data processing method

**Fig. 1:**
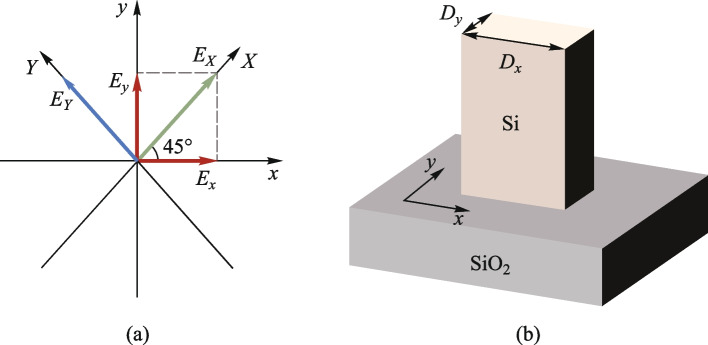
**a** Two sets of perpendicular coordinate axes, labeled as *x* and *y*, and *X* and *Y*, are situated in the same plane, with an angle of 45° between the *x* and *X* axes. Both ${E}_{X}$ and ${E}_{Y}$ can be expressed as ${E}_{x}$ and ${E}_{y}$; **b** Depicts a unit structure capable of independently controlling the phase of the *x* and *y* components of the electric field, with a phase modulation range from 0 to 2π

When constructing the router model, it involves two degrees of freedom: polarization and wavelength. The complex amplitude of the linearly polarized light electric field can be represented by the following equation:

1 $$\begin{aligned} E=\left(\genfrac{}{}{0pt}{}{{E}_{x}}{{E}_{y}}\right)={E}_{0}\left(\genfrac{}{}{0pt}{}{\text{exp}\left(\text{j}{\varphi }_{1}\right)}{\text{exp}\left(\text{j}{\varphi }_{2}\right)}\right),\end{aligned}$$

where ${\text{j}}^{2}=-1$, and ${E}_{x}$ and ${E}_{y}$ represent the *x* and *y* components of the electric field, ${\varphi }_{1}$ and ${\varphi }_{2}$ represent the phase of the *x* and *y* components of the electric field, respectively. In Eq. (1), ${E}_{x}$ and ${E}_{y}$ have equal amplitudes, both set to 1, denoted as *E*_0_. When ${\varphi }_{1}={\varphi }_{2}$, ${E}_{x}$ and ${E}_{y}$ combine to form light polarized in the *X* direction, denoted as ${E}_{X}$, as shown by the green line in Fig. 1a. When ${|\varphi }_{1}-{\varphi }_{2}|=\uppi$, ${E}_{x}$ and ${E}_{y}$ combine to form light polarized in the *Y* direction, denoted as ${E}_{Y}$, depicted by the blue line in Fig. 1a. The input ${E}_{X}$ or ${E}_{Y}$ can be represented as a superposition of ${E}_{x}$ and ${E}_{y}$ with certain phase differences.

Our data set comprises one dimension describing polarization and one dimension describing wavelength. The polarization is described by two channels, and the wavelength dimension is divided into multiple channels. The two polarization channels are denoted as Channel 1 and Channel 2, where the data in Channel 1 describe the complex amplitude of ${E}_{x}$, and the data in Channel 2 describe the complex amplitude of ${E}_{y}$. Different wavelength channels represent corresponding wavelengths.

**Fig. 2:**
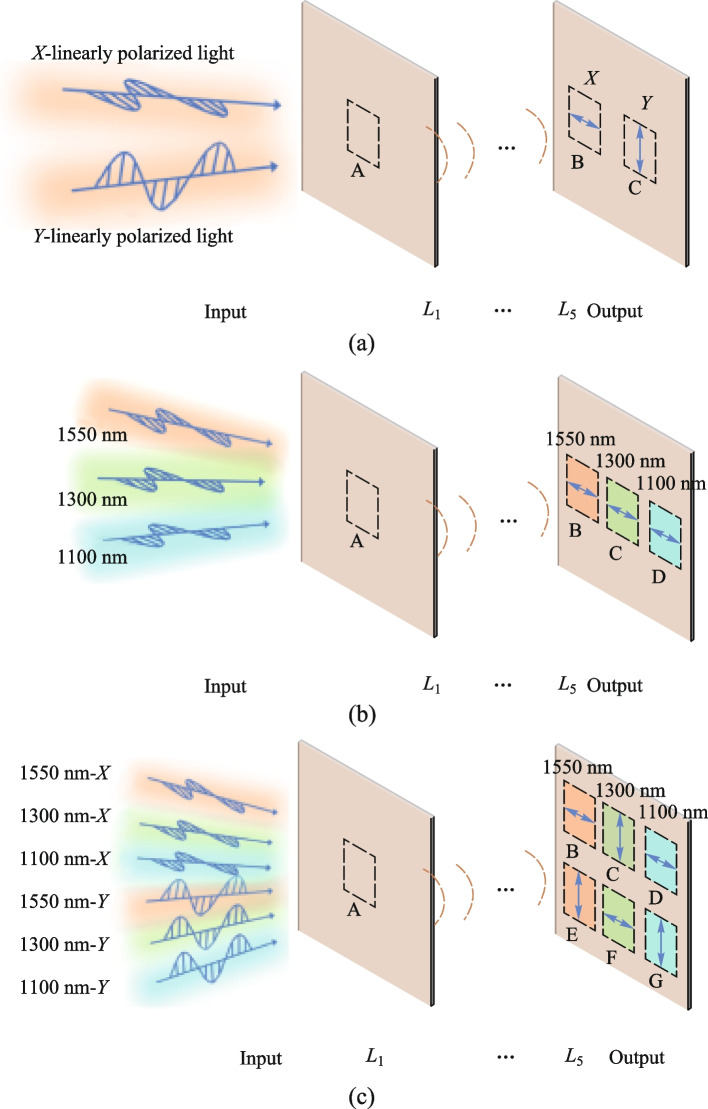
**a** Schematic diagram of polarization router. **b** Schematic diagram of wavelength router. **c** Schematic diagram of polarization-wavelength composite router

The unit structures employed in our model, as illustrated in Fig. 1b, have the capability to independently influence both the *x* and *y* components of the electric field. Precise modulation of the transmitted phase within the 0 to 2π range is achieved by adjusting the *D*_*x*_ and *D*_*y*_ of the Si units (See Supplementary Note 1). Each unit serves as a neuron, enabling the manipulation of optical signals within the neural network. In our D^2^NN, the input layer is designated as *L*_0_, followed by five diffractive layers denoted as *L*_1_ through *L*_5_ in Fig. 2. Each diffractive layer is composed of neurons.

Figure 2 illustrates the overall process of implementing routers in D^2^NN. Different types of polarized light or electric fields with varying wavelengths are input at port A and processed through the diffractive layers. This results in output at different ports, enabling polarization or wavelength routing. Since the devices we designed serve as routers, each device outputs signals only at the target port corresponding to the different types of inputs, and the characteristics of their expected outputs’ electric fields are identical to those of the input light. For example, in the case of the polarization-wavelength composite router, when the input is *X*-linearly polarized light with a wavelength of 1550 nm, its expected output is the 1550 nm *X*-polarized light with the same electric field intensity as the input light, which will be output at target port B. Meanwhile, the signals input at the ports are all uniform signals.

### Loss function

For each router, every input signal ideally results in an output at the specified location, termed the target output value. The computed output from the model is referred to as the actual output value. To quantify the difference between the actual and target output values during learning, we use the classical mean absolute error (MAE) loss function, denoted as ${L}_{\text{MAE}}$, expressed as follows:

2 $$\begin{array}{c}{L}_{\text{MAE}}=\frac{1}{N}{\sum }_{i}^{N}|{E}_{i}-{G}_{i}|,\end{array}$$

where *N* represents the number of diffractive units in the output layer, which is 50 × 50, *E*_*i*_ represents the target output value, and *G*_*i*_ represents the actual output value.

MAE is widely employed as a loss function in diffractive neural networks, offering advantages over alternatives such as mean squared error. Its robustness to outliers stems from its focus on absolute differences rather than squared differences. Additionally, MAE disregards error direction, simplifying interpretation and model evaluation. Lower MAE values indicate better agreement between target and actual values. In summary, the MAE plays a pivotal role in quantifying the discrepancies within our diffractive neural networks.

### Training process

**Fig. 3:**
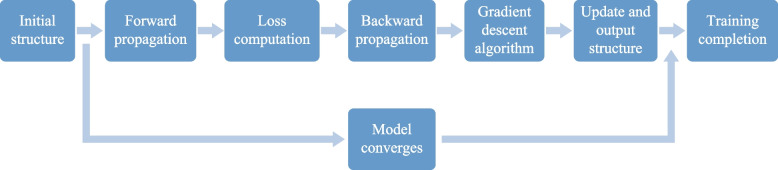
Flowchart of the neural network training process

Figure 3 illustrates the training process of the neural network, involving iterative optimization aimed at adjusting network parameters to minimize the loss function and enhance model performance. Initially, the initial structure of the model is defined. In this study, the D^2^NN framework consists of an input layer and five diffractive layers, with the last diffractive layer also serving as the output layer. Each diffractive layer is a square with a side length of 41.075 μm, and each layer contains 50 × 50 diffractive elements. The distance between adjacent diffractive layers is 387.5 μm. Subsequently, training data are fed into the network through forward propagation to generate predicted outputs. Then, the loss function is computed. Following this, backpropagation is performed to calculate the derivatives of the loss function with respect to the neural network parameters. This allows for the utilization of gradient descent to update the network parameters, continuously adjusting the transmission amplitude and phase of the incident electric field by modifying the sizes of the diffraction elements *D*_*x*_ and *D*_*y*_, thereby minimizing the error between the actual and target output values. The iterative process comprises forward propagation, loss computation, backpropagation, and parameter updates, repeated for a predetermined number of training iterations. In regard to the specific principles of the propagation model, please refer to Supplementary Note 2. Finally, the model is evaluated and refined to achieve optimal training results.

## Results and discussion

### Polarization router

Initially, we implemented a polarization router capable of routing both *X*-linearly polarized light and *Y*-linearly polarized light. As depicted in Fig. 2a, when *X*-linearly polarized light is input at port A, the electric field is output at port B, while *Y*-linearly polarized light input at port A results in the electric field being output at port C. The amplitude of ${E}_{X}$ or ${E}_{Y}$ is $\sqrt{2}$, and the output at port B or C is defined as ${E}_{\text{out}}$. Ideally, the amplitude of ${E}_{\text{out}}$ is $\sqrt{2}$. Each port is divided into two polarization channels: Channel 1 represents the *x* component of the electric field, and Channel 2 represents the *y* component. Thus, ideally, the intensity of ${E}_{\text{out}}$ in each polarization channel is 1.

**Fig. 4:**
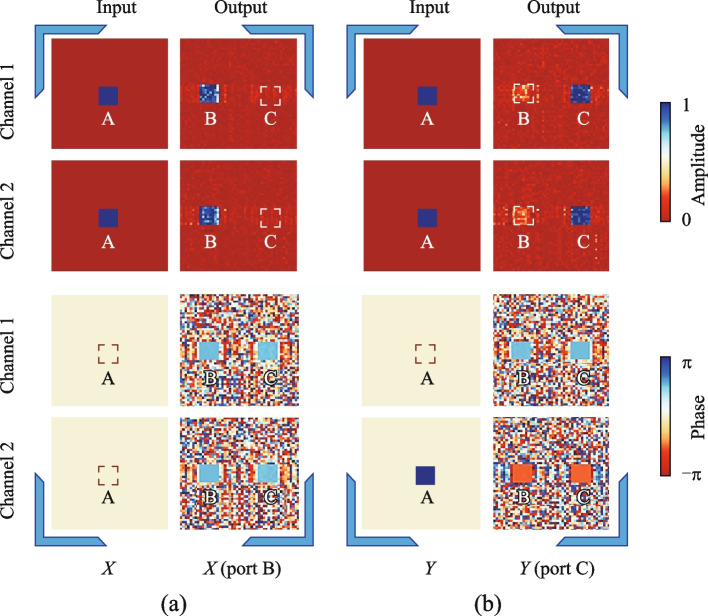
Testing results of the polarization router. **a** When the input is *X*-linearly polarized light, the amplitude and phase distribution of the input and output optical fields in Channels 1 and 2, resulting in the output of *X*-linearly polarized light at port B. **b** When the input is *Y*-polarized light, the amplitude and phase distribution of the input and output electric fields in Channels 1 and 2, resulting in the output of *Y*-linearly polarized light at port C

The field distribution modulated by the trained diffractive network is illustrated in Fig. 4. In Fig. 4a, the electric field amplitudes of Channel 1 and Channel 2 at input port A are both 1, with phases of 0, indicating inputting *X*-linearly polarized light. After modulation by the trained diffractive network, at output port B, the electric field amplitudes of both Channel 1 and Channel 2 are close to 1, with phases approximately π/2, indicating the output is *X*-linearly polarized light. Meanwhile, output port C exhibits almost no optical power output. Similarly, in Fig. 4b, the electric field amplitudes of Channel 1 and Channel 2 at port A are both 1, but with Channel 1 having a phase of 0 and Channel 2 having a phase of π, indicating inputting *Y*-linearly polarized light. After processing through the five diffractive layers, at output port C, the electric field amplitudes of both Channel 1 and Channel 2 are close to 1. Channel 1 has a phase of π/2, while Channel 2 has a phase of − π/2, resulting in a phase difference of π between the two channels, indicating output *Y*-linearly polarized light. This demonstrates the effective performance of our diffractive network in carrying out the given tasks.

The insertion loss (IL) represents the efficiency of light signal transmission in the device and can be calculated by the ratio of the optical intensity at the output port to the total input optical intensity:

3 $$\begin{array}{c}IL=-10{\text{log}}_{10}\left(\frac{{I}_{\text{out}}}{{I}_{\text{in}}}\right),\end{array}$$

where ${I}_{\text{out}}$ is the total input optical intensity, and in the calculation process, the optical intensity of each input type is the same. A lower insertion loss value indicates a higher efficiency of light signal transmission in the device. Each computation of the router only outputs a signal at one port, while the outputs at other ports are noise. We use the extinction ratio (ER) to denote the distinction in optical intensity between the signal light and noise. The calculation formula is:

4 $$\begin{array}{c}ER=10{\text{log}}_{10}\left(\frac{{I}_{\text{signal}}}{{I}_{\text{noise}}}\right),\end{array}$$

where ${I}_{\text{signal}}$ represents the optical intensity of ${E}_{\text{out}}$, and ${I}_{\text{noise}}$ represents the optical intensity of the non-signal output ports. For a well-trained polarization router, the output performance is similar when *X*-linearly polarized light and *Y*-linearly polarized light are input. This characteristic also applies to routers with other degrees of freedom. Therefore, for convenience, we use the average output performance corresponding to these two input types to represent the performance of the respective router. For a well-trained polarization router, its insertion loss is 1.43 dB, and the extinction ratio is 7.22 dB. The high accuracy of the output results can also be observed in Fig. 4.

In all the routers mentioned below, the input configuration is similar to the polarization router. The amplitude of the electric field in each input port is $\sqrt{2}$, resulting in an amplitude of 1 for each channel. Additionally, if the input is *X*-linearly polarized light, the phases of Channel 1 and Channel 2 are the same. Conversely, if the input is *Y*-linearly polarized light, the phase difference between Channel 1 and Channel 2 is π. Further details on the input configurations of other routers will not be elaborated in the subsequent sections.

### Wavelength router

In addition to leveraging the polarization degree of freedom, we also utilized the wavelength degree of freedom to implement wavelength routers. These routers can route two or even three wavelengths and perform tasks exceptionally well. Here, we introduce an additional data dimension, segmented based on wavelength into different channels, similar to the polarization channels mentioned earlier. Each wavelength channel corresponds to a specific wavelength, meaning the electric field values for each wavelength exist only within their corresponding channel.

First, we implemented two two-input wavelength routers capable of routing electric fields with wavelengths of 1300 and 1550 nm while maintaining polarization (See Supplementary Note 3). To expand the capability of the wavelength router, we further attempted and achieved a device capable of routing three wavelengths. Under the same polarization conditions, this three-input wavelength router can route electric fields with wavelengths of 1100, 1300, and 1550 nm.

Under *X*-linearly polarized light, the three-input wavelength router is depicted in Fig. 2b. When light with wavelengths of 1550, 1300, or 1100 nm is input at port A, it undergoes propagation processing by the diffractive layers and is respectively output at port B, C, or D.

**Fig. 5:**
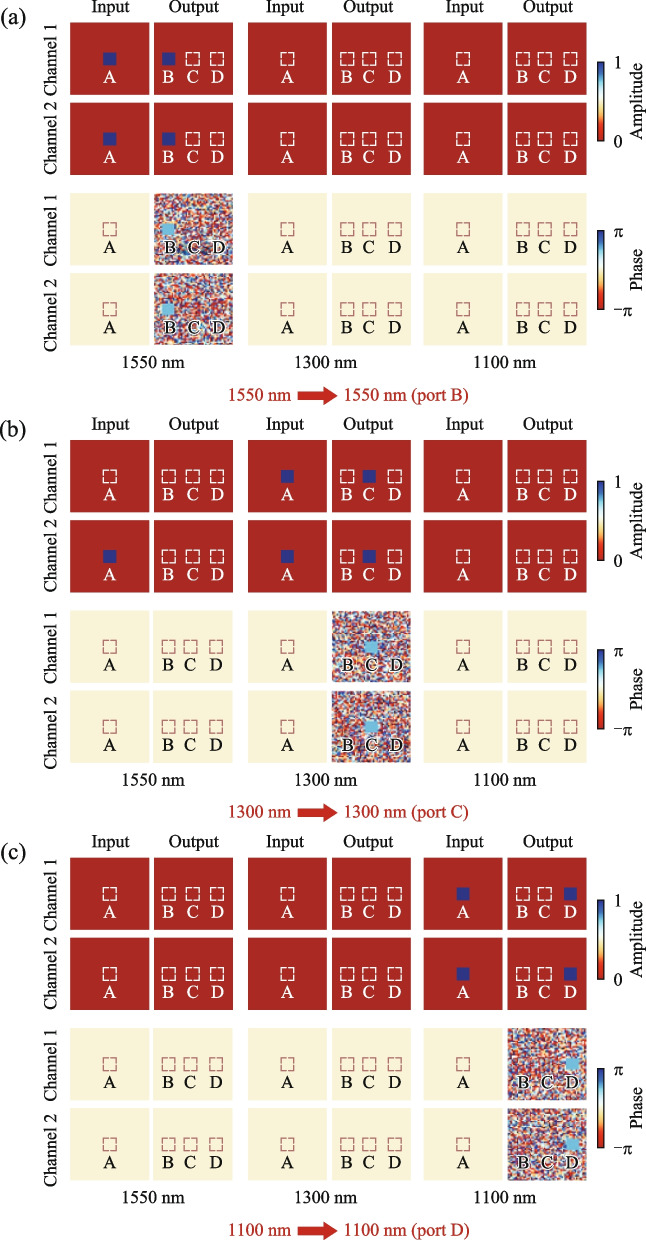
Training results of wavelength router under *X*-linearly polarized light. The images demonstrate the amplitude and phase distribution of the input and output electric fields in the polarization and wavelength channels. Specifically, it depicts the scenario when the input light has a wavelength of 1550 nm **a**, 1300 nm **b**, or 1100 nm **c**. The final electric field is output at ports B **a**, C **b**, or D **c**, respectively

As illustrated in Fig. 5a, when *X*-linearly polarized light with a wavelength of 1550 nm is input, there is no electric field data in the 1300 and 1100 nm channels. In the 1550 nm channel, the electric field amplitudes in Channel 1 and Channel 2 at input port A are both 1, with phases of 0. Hence, the input electric field is *X*-linearly polarized light with a wavelength of 1550 nm. After propagating through the diffractive layers, the electric field amplitudes in Channel 1 and Channel 2 at output port B are close to 1, with phases of π/2, resulting in *X*-linearly polarized light. In Fig. 5b, when *X*-linearly polarized light with a wavelength of 1300 nm is input, the electric field data are only present in the 1300 nm channel. After propagation through the diffractive layers, the electric field amplitudes in Channel 1 and Channel 2 at output port C are both close to 1, with phases of π/2, resulting in *X*-linearly polarized light. Similarly, in Fig. 5c, when *X*-linearly polarized light with a wavelength of 1100 nm is input, the electric field data are only present in the 1100 nm channel. After propagating through the diffractive layers, the electric field amplitudes in Channel 1 and Channel 2 at output port D are both close to 1, with phases of π/2, resulting in *X*-linearly polarized light. The insertion loss of the trained wavelength router is 0.013 dB, and the extinction ratio reaches 18.96 dB.

The computation process for the three-input wavelength router under *Y*-linearly polarized light differs only in terms of polarization from that under *X*-linearly polarized light. The computed results of the three-input wavelength router under *Y*-linearly polarized light closely resemble those under *X*-linearly polarized light (See Supplementary Note 4). Additionally, the output signal efficiency of the three-input wavelength router is exceptionally high, exhibiting significant differentiation between the signal and noise. Thus, the performance of the three-input wavelength router is outstanding.

### Polarization-wavelength composite router

In the preceding sections, we have individually designed routers targeting polarization and wavelength, with the wavelength router exhibiting superior performance compared to the polarization router. To enhance the capacity of information processing, we propose routers capable of simultaneously handling polarization and wavelength. These routers are trained to handle four (See Supplementary Note 5) and six different electric field inputs, respectively. In the six-input polarization-wavelength composite router, the six-input electric fields comprise *X*-linearly polarized light with wavelengths of 1550, 1300, and 1100 nm, as well as *Y*-linearly polarized light with wavelengths of 1550, 1300, and 1100 nm.

Figure 2c illustrates the schematic diagram of the six-input polarization-wavelength composite router, with port A designated as the input port and ports B, C, D, E, F, and G as the output ports. Port A can input six types of electric fields. When *X*-linearly polarized light with wavelengths of 1550, 1300, or 1100 nm is input at port A, it is modulated by the diffractive layer and output at ports B, F, or D, respectively. When *Y*-linearly polarized light with wavelengths of 1550, 1300, or 1100 nm is input at port A, it is modulated by the diffractive layer and output at ports E, C, or G, respectively.

**Fig. 6:**
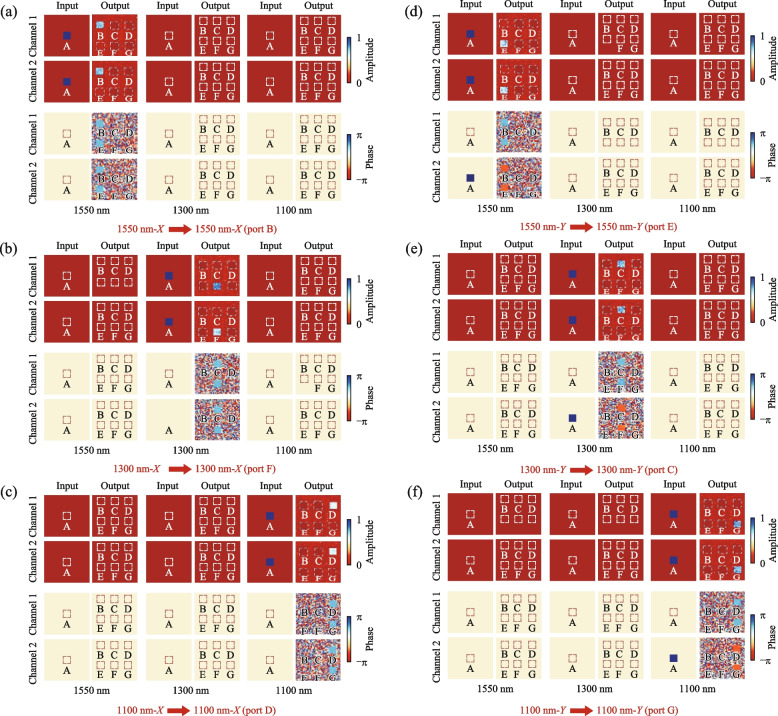
Training results of the polarization-wavelength composite router. The images demonstrate the amplitude and phase distribution of the input and output electric fields in the polarization and wavelength channels. Specifically, it depicts the scenario when *X*-linearly polarized light with wavelengths of 1550 nm **a**, 1300 nm **b**, or 1100 nm **c**, or *Y*-linearly polarized light with wavelengths of 1550 nm **d**, 1300 nm **e**, or 1100 nm **f** is input at port A, it is output at ports B **a**, F **b**, D **c**, E **d**, C **e**, or G **f**, respectively

When *X*-linearly polarized light with a wavelength of 1550 nm is input at port A, as shown in Fig. 6a, there is no electric field in the 1300 and 1100 nm channels. In the 1550 nm channel, the electric field amplitude for both Channels 1 and 2 at input port A is 1, with phases of 0. Therefore, the input electric field is *X*-linearly polarized light with a wavelength of 1550 nm. After propagating through the diffractive layers, the electric field is ultimately output at port B, with both Channels 1 and 2 having electric field amplitudes close to 1 and phases around π/2, resulting in *X*-linearly polarized light. Figure 6b illustrates the electric field distribution in each channel when the input is *X*-linearly polarized light at 1300 nm, with the electric field ultimately output at port F as *X*-linearly polarized light. Figure 6c displays the electric field distribution in each channel when the input is *X*-linearly polarized light at 1100 nm, with the electric field ultimately output at port D as *X*-linearly polarized light. When *Y*-linearly polarized light with a wavelength of 1550 nm is input at port A, as depicted in Fig. 6d, the electric field data are only present in the 1550 nm channel. After propagating through the diffractive layers, the electric field is output at port E, with both Channels 1 and 2 having electric field amplitudes close to 1. The phase in Channel 1 is π/2, while the phase in Channel 2 is − π/2, resulting in *Y*-linearly polarized light. When *Y*-linearly polarized light with a wavelength of 1300 nm is input at port A, as illustrated in Fig. 6e, the electric field data are only present in the 1300 nm channel. The electric field amplitude at input port A for both Channels 1 and 2 is 1, with a phase of 0 in Channel 1 and a phase of π in Channel 2. After propagating through the diffractive layers, the electric field with a wavelength of 1300 nm is ultimately output at port C, with both Channels 1 and 2 having electric field amplitudes close to 1. The phase in Channel 1 is π/2, while the phase in Channel 2 is − π/2, resulting in *Y*-linearly polarized light. Finally, when *Y*-linearly polarized light with a wavelength of 1100 nm is input at port A, as shown in Fig. 6f, the electric field is ultimately output at port G as *Y*-linearly polarized light.

After training, the wavelength router exhibits an insertion loss of 2.09 dB, and an extinction ratio of 11.16 dB. Compared to the previously designed polarization router and wavelength router, the overall performance of the polarization-wavelength composite router slightly declines. This is primarily attributed to the increased complexity of tuning degrees of freedom, sacrificing signal-to-noise ratio for higher information capacity. These findings demonstrate the versatility and potential of our devices in advancing complex optical information processing tasks.

## Conclusion

The D^2^NN based on polarization and wavelength encoding in our research corresponds to pixel sizes at the several hundreds of nanometer level. The all-optical router based on this D^2^NN can be fabricated using micro/nano processing technology compatible with Complementary metal oxide semiconductor (CMOS) technology, as the current state-of-the-art e-beam lithography (EBL) technology has a processing resolution of only a few nanometers. The device structure in our research is based on a two-dimensional diffraction layer. The integration process necessitates stacking and connecting diffraction multiple layers within the chip plane, allowing signal propagation in the direction perpendicular to this plane. This process may involve challenges, including issues related to overlap, alignment, and other aspects [54, 55]. Transforming this diffractive layer into a one-dimensional structure, essentially creating a device for in-plane signal propagation on the chip, would reduce system complexity and enhance integration—a pathway we find worth exploring.

Overall, our study successfully implemented various routers based on the D^2^NN computing framework, including the polarization router capable of routing two orthogonally polarized light beams, the wavelength router, and the polarization-wavelength composite router. The polarization-wavelength composite router can route six types of input light, which are formed by pairwise combinations of three wavelengths (1550, 1300, and 1100 nm) and two orthogonal linearly polarized lights, enhancing the information processing capability of the device. The wavelength router effectively routes light at wavelengths of 1550, 1300, and 1100 nm. It demonstrates exceptional performance, featuring an insertion loss as low as 0.013 dB and an extinction ratio of up to 18.96 dB. These results underscore the strong tuning capabilities of the D^2^NN framework for light with different wavelengths. In future research, to further enhance the performance of the router, we can incorporate nonlinear operators into the design of the diffractive layer. This will improve the tuning capabilities of the neural network, enabling it to route a greater variety of input electric fields. Additionally, algorithmic optimizations during the initial training phases and the incorporation of inverse design techniques at the device design stage can also improve device performance. Future studies could expand the wavelength router to support n wavelength channels and integrate them with two polarization channels, thereby increasing the total information channels to 2*n*. This approach would significantly enhance the capacity for processing information. In addition to these methods, we can also optimize the D^2^NN architecture by exploring more complex algorithms, enabling the device to handle elliptical polarization states and thus expanding its applicability in real-world scenarios. In summary, we have successfully introduced polarization and wavelength degrees of freedom into the D^2^NN framework to achieve routing. These routers exhibit passive, low-loss, and high-extinction-ratio characteristics, highlighting the effectiveness and versatility of D^2^NN-based routers. This study paves the way for the future implementation of all-optical routers and promotes the advancement of optical computing.

### Supplementary Information

Supplementary Material 1.

## Data Availability

Data underlying the results presented in this paper are not publicly available at this time but may be obtained from the authors upon reasonable request.
