# Supplementary material for: Polarization and wavelength routers based on diffractive neural network
Source: Front Optoelectron. 2024 Jul 16;17(1):22. doi: 10.1007/s12200-024-00126-2 (PMC11250754; doi:10.1007/s12200-024-00126-2)
Supplement: Supplementary file 1 — Supplementary Material 1. [file 12200_2024_126_MOESM1_ESM.pdf]

Supplementary Information

Polarization and wavelength routers based on diffractive neural network

Xiaohong Lin,[1] Yulan Fu,[1,*] Kuo Zhang,[2] Xinping Zhang,[1] Shuai Feng,[2] and Xiaoyong Hu[3, 4, 5,*]

[1]*School of Physics and Optoelectronic Engineering, Beijing University of Technology, Beijing 100124, P. R. China*
[2]*School of Science, Minzu University of China, Beijing100081, China*
[3]*State Key Laboratory for Mesoscopic Physics and Department of Physics, Collaborative Innovation Center of Quantum Matter, Beijing Academy of Quantum Information Sciences, Nano-optoelectronics Frontier Center of Ministry of Education, Peking University, Beijing100871, China*
[4]*Collaborative Innovation Center of Extreme Optics, Shanxi University, Taiyuan, Shanxi030006, China*
[5]*Peking University Yangtze Delta Institute of Optoelectronics, Nantong, Jiangsu226010, China*
*fuyl@bjut.edu.cn, xiaoyonghu@pku.edu.cn

Supplementary Note 1: Simulation of the unit structure

Fig. S1(a) shows the unit structure used in the manuscript, with a fixed height H. Fig. S3(a) shows the variation of the real and imaginary parts of the refractive index of the Si substrate with wavelength, as used in the calculations. When linearly polarized light is incident along the corresponding axis, the nanopillar produces a polarization-dependent phase shift, which can be expressed as a function of D_x and D_y. The phase and amplitude under x-linearly polarized light and y-linearly polarized light are simulated by the finite-difference time-domain (FDTD) method (Fig. S1(b)), it can be observed that this structure achieves very high transmittance (close to 100% within the simulation range) and a phase distribution of 0-2π. The wavelength is chosen to be 1550 nm, with a nanopillar period of 600 nm and a height of H = 800 nm. With the same parameters, the simulation results for incident light at wavelengths of 1100 nm and 1300 nm exhibit similar trends to those observed at 1550 nm (Fig. S2).

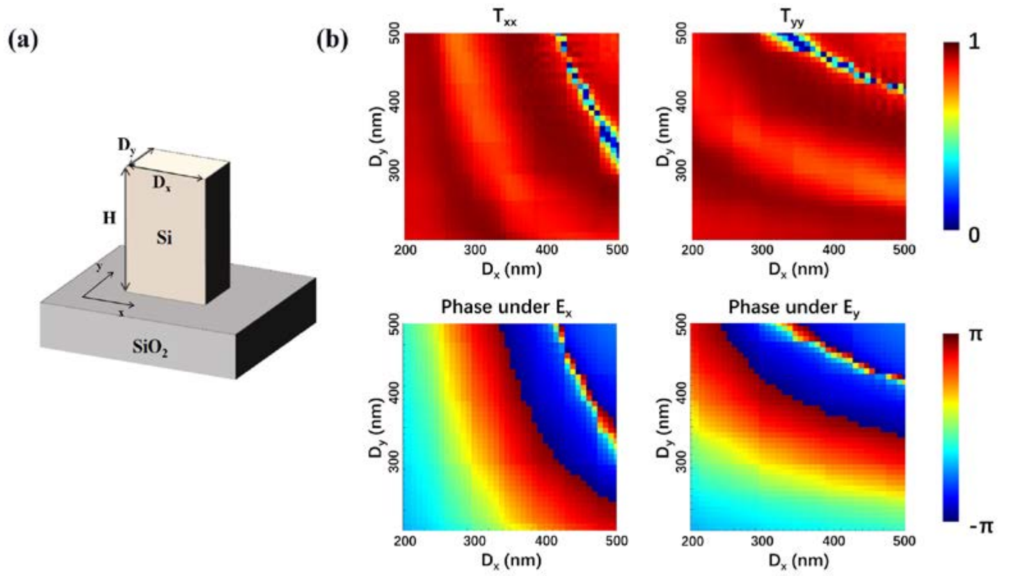

Fig. S1 (a) Unit structure with a fixed height H and adjustable structural dimensions D_x and D_y; (b) Simulated transmission coefficients (T_{xx}, T_{yy}) and phase shifts (φ_{xx}, φ_{yy}) under x-linearly and y-linearly polarized light with a wavelength of 1550 nm. The nanopillar period is 600 nm and the height H is 800 nm

To validate the practicality of the proposed approach, while keeping other parameters constant, we increased the thickness H of the Si substrate to 1000 nm. This relatively higher thickness can be exposed by a thicker resist as well as high-pressure EBL. Fig. S3(b) shows the simulated transmission coefficients and phase shifts under x-linearly and y-linearly polarized light when H is 1000 nm in the unit structure. The incident wavelength is 1550 nm, and the nanopillar period is 600 nm. With the same parameters, the simulation results for incident light at wavelengths of 1100 nm and 1300 nm exhibit similar trends to those observed at 1550 nm (Fig. S4). It can be observed that this structure also achieves very high transmittance (close to 100% within the simulation range) and a phase distribution of 0-2π. It also exhibits significant orthogonality variability, indicating that nanopillars can be effectively applied in multilayer structures.

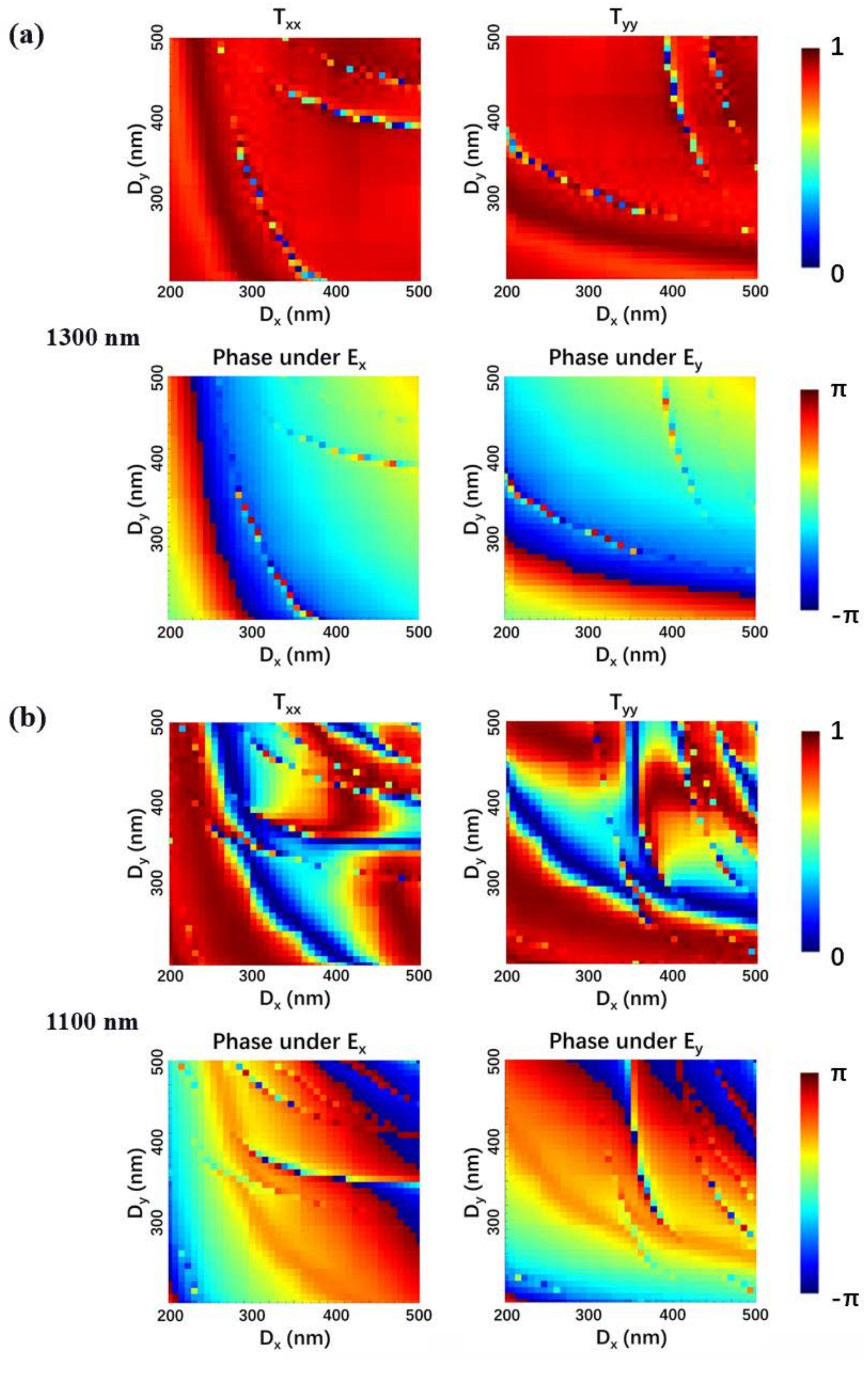

Fig. S2 Simulated transmission coefficients (T_{xx}, T_{yy}) and phase shifts (φ_{xx}, φ_{yy}) under x-linearly and y-linearly polarized light with wavelengths of 1300 nm (a) and 1100 nm (b). The nanopillar period is 600 nm and the height H is 800 nm

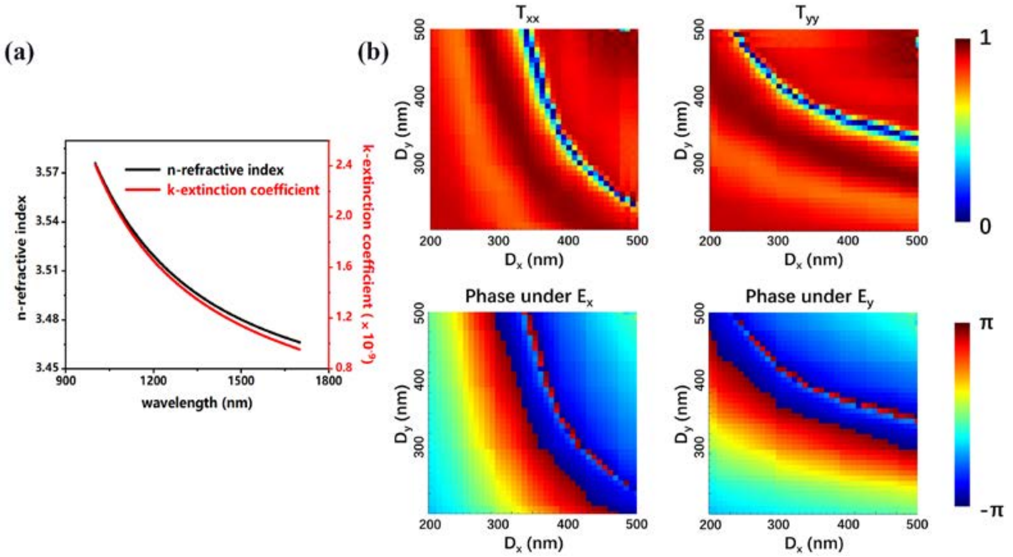

Fig. S3 (a) The real and imaginary parts of the refractive index of the Si substrate as a function of wavelength. (b) Simulated transmission coefficients (T_{xx}, T_{yy}) and phase shifts (φ_{xx}, φ_{yy}) under x-linearly and y-linearly polarized light with a wavelength of 1100 nm and 1300 nm. The nanopillar period is 600 nm and the height H is 1000 nm

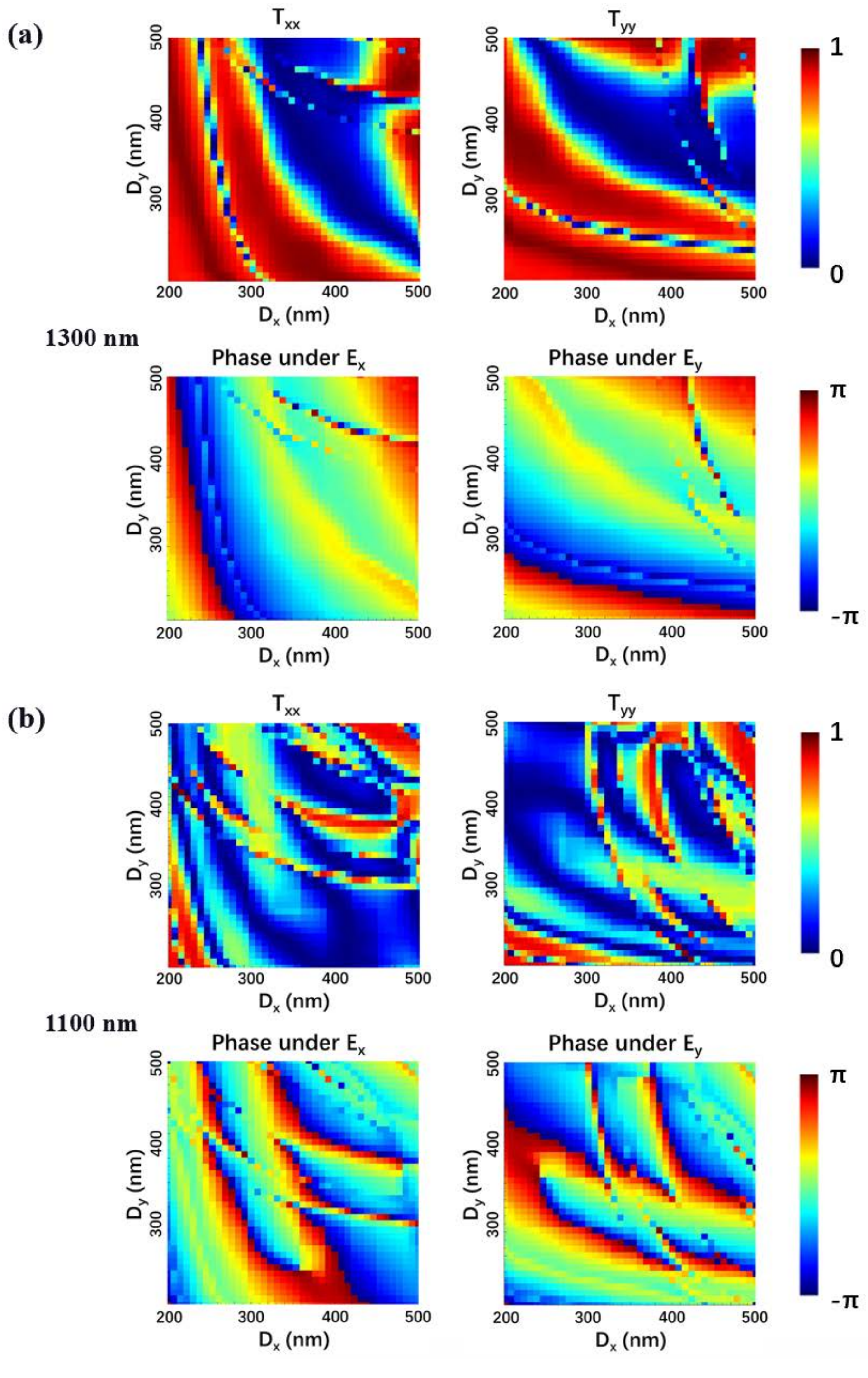

Fig. S4 Simulated transmission coefficients (T_{xx}, T_{yy}) and phase shifts (φ_{xx}, φ_{yy}) under x-linearly and y-linearly polarized light with wavelengths of 1300 nm (a) and 1100 nm (b). The nanopillar period is 600 nm and the height H is 1000 nm

Supplementary Note 2: Propagation model

The optical diffraction connects each neuron to others in the subsequent layers. Following the principles of Rayleigh-Sommerfeld diffraction, each diffractive unit/neuron can be regarded as the coherent superposition of the propagated light from each diffractive unit/neuron in the previous layer. It can also be interpreted as the source of secondary waves, fully connected to the next layer. The equation for light propagation between diffractive layers is expressed as follows:

$$w_i^l(x, y, z) = \frac{z - z_i}{r^2}\left(\frac{1}{2\pi r} + \frac{1}{\mathrm{j}\lambda}\right)\exp\left(\frac{\mathrm{j}2\pi r}{\lambda}\right), \tag{1}$$

where r is defined as the distance between the coordinates (x, y, z) of a point and the coordinates (x_i, y_i, z_i) of the *i*-th diffractive unit in the *l*-th layer, given by:

$$r = \sqrt{(x - x_i)^2 + (y - y_i)^2 + (z - z_i)^2}. \tag{2}$$

The notation $w_i^l(x, y, z)$ in Eq. (1) represents the complex-valued field that propagated to each diffractive unit located at (x, y, z) in the (*l*+1)-th layer. This propagation is achieved by utilizing the *i*-th diffractive unit located at (x_i, y_i, z_i) in the *l*-th layer as the wave source, with a wavelength of λ, which is 1550 nm. We use u_i^l to represent the light field function of the *l*-th layer's *i*-th neuron during the propagation process, and it can be expressed as:

$$u_i^l(x_i, y_i, z_i) = \sum_{j \in N} u_j^{l-1}(x_i, y_i, z_i) \cdot t^l(x_i, y_i, z_i) \cdot w_i^{l-1}(x_i, y_i, z_i), \tag{3}$$

where *N* represents the number of diffractive units/neurons on the diffractive layer. The notation $t^l(x_i, y_i, z_i)$ represents the modulation of the optical field complex amplitude by the lth diffractive layer and can be expressed using the following equation:

$$t^l(x_i, y_i, z_i) = a^l(x_i, y_i, z_i) \cdot \exp[\mathrm{j}\phi^l(x_i, y_i, z_i)], \tag{4}$$

where the parameters a^l and ϕ^l represent the amplitude and phase coefficients, respectively, at the (x_i, y_i, z_i) coordinates of the l-th layer. These two parameters can be continuously adjusted through deep learning. Each neuron's transmitted amplitude and transmitted phase values in the diffractive network are optimized during training.

Considering the substantial computational burden of solving the conventional D^2NN model using the Rayleigh-Sommerfeld diffraction formula, we utilize Fresnel scalar diffraction to reduce the computational effort. Given the specific layer spacing conditions under consideration, the Rayleigh-Sommerfeld diffraction formula can be interchangeably employed with the Fresnel scalar diffraction theory in the obtained results. Here, we employ the Fresnel scalar diffraction theory to construct the forward propagation model of our diffractive neural network. This approach not only reduces computational burden but also enables more efficient and accurate modeling of the beam's complex amplitude throughout the network. The complex amplitude u_i^l of the beam for the l-th layer's i-th neuron can be expressed as follows:

$$u_i^l(x_i, y_i) = \mathrm{F}^{-1}\left\{\mathrm{F}\left[u_i^{l-1}(x_i, y_i) \cdot t^{l-1}(x_i, y_i) \cdot H\left(f_x, f_y\right)\right]\right\}. \quad (5)$$

Here $H\left(f_x, f_y\right)$ is the transformation function in the frequency domain during beam propagation, given by:

$$H\left(f_x, f_y\right) = \exp[\mathrm{j}k(z - z_i)] \cdot \exp\left[-\mathrm{j}\lambda\pi(z - z_i)\left(f_x^2 + f_y^2\right)\right], \quad (6)$$

where $k = 2\pi/\lambda$ representing the wave number. In Eq. (5), F and F^{-1} represent the fast Fourier transform and inverse fast Fourier transform, respectively.

The training process of D^2NN primarily utilizes methods such as backpropagation and gradient descent to optimize results.

Supplementary Note 3: Two-input wavelength router

We implemented two two-input wavelength routers capable of routing electric fields with wavelengths of 1300 nm and 1550 nm while maintaining polarization. Under the same polarization conditions, the two-input wavelength router can route electric fields with wavelengths of 1550 nm and 1300 nm.

Under X-linearly polarized light, the three-input wavelength router is depicted in Fig. S5(a). Port A can input light with wavelengths of 1550 nm or 1300 nm. When light with wavelengths of 1550 nm, or 1300 nm is input at port A, it undergoes propagation processing by the diffractive layers and is respectively output at ports B, or C.

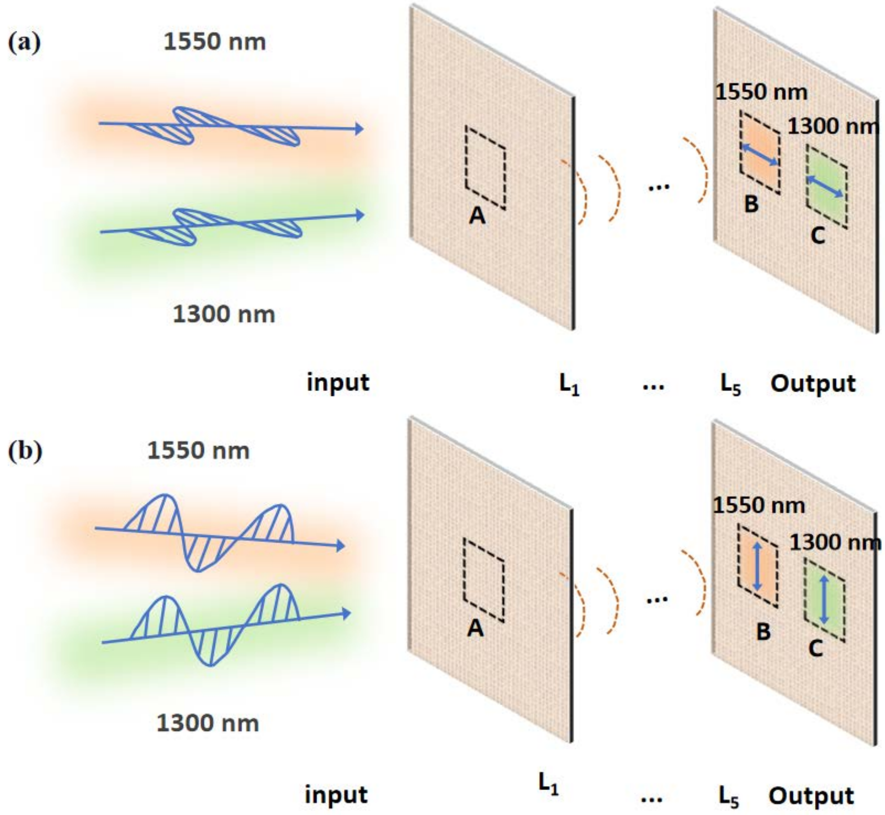

Fig. S5 Schematic diagram of two-input wavelength router. (a) and (b) depict the cases when the electric field is X-linearly polarized light and Y-linearly polarized light, respectively. A represents the input port of the input layer, while B and C represent the two output ports located at the output layer

As illustrated in Fig. S6(a), when X-linearly polarized light with a wavelength of 1550 nm is input, there is no electric field data in the 1300 nm channel. In the 1550 nm channel, the electric field amplitudes in Channel 1 and Channel 2 at input port A are both 1, with phases of 0. Hence, the input electric field is X-linearly polarized light with a wavelength of 1550 nm. After propagating through the diffractive layers, the electric field amplitudes in Channel 1 and Channel 2 at output port B are close to 1, with phases of π/2, resulting in X-linearly polarized light. In Fig. S6(b), when X-linearly polarized light with a wavelength of 1300 nm is input, only the 1300 nm channel has electric field data. After propagation through the diffractive layers, the electric field amplitudes in Channel 1 and Channel 2 at output port C are both close to 1, with phases of π/2, resulting in X-linearly polarized light. The insertion loss of the trained wavelength router is 0.01 dB, and the extinction ratio reaches 25.39 dB.

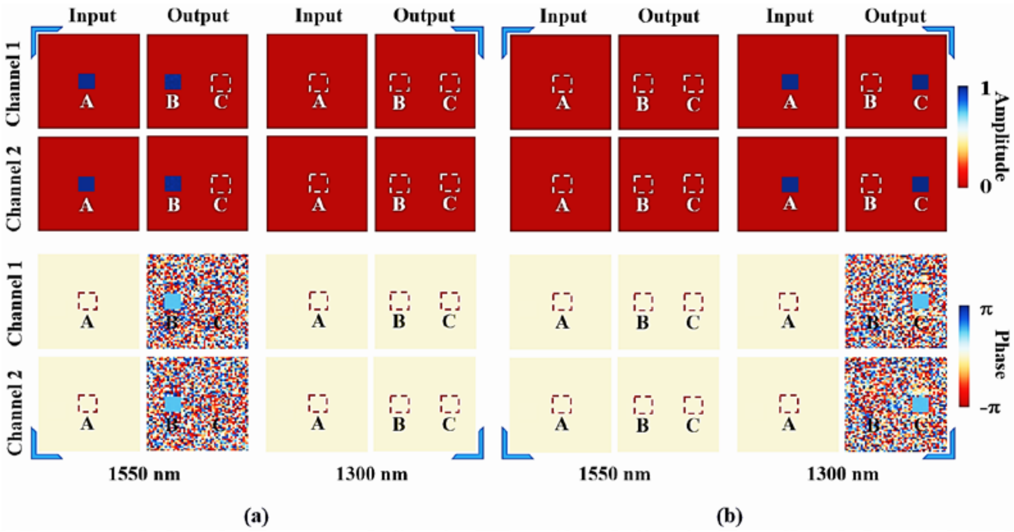

Fig. S6 The training results of the two-input wavelength router under X-linearly polarized light. The images demonstrate the amplitude and phase distribution of the input and output electric fields in the polarization and wavelength channels. Specifically, it depicts the scenario when the input light has a wavelength of 1550 nm (a), or 1300 nm (b). The final electric field is output at ports B (a), or C (b), respectively

In the case of Y-linearly polarized light, the wavelength router is depicted in Fig. S5(b). The computational process is similar to that under X-linearly polarized light, with the only difference being the polarization. When Y-linearly polarized light with a wavelength of 1550 nm is input, as shown in Fig. S7(a), the final electric field is output at port B. Similarly, when Y-linearly polarized light with a wavelength of 1300 nm is input, as illustrated in Fig. S7(b), the final electric field is output at port C. This router exhibits a low insertion loss of 0.004 dB, crosstalk of -22.52 dB, and achieves an extinction ratio of 22.51 dB, which closely resembles its performance under X-linearly polarized light. Moreover, it demonstrates high output signal efficiency and significant differentiation between signal and noise, indicating excellent performance of the two-input wavelength router.

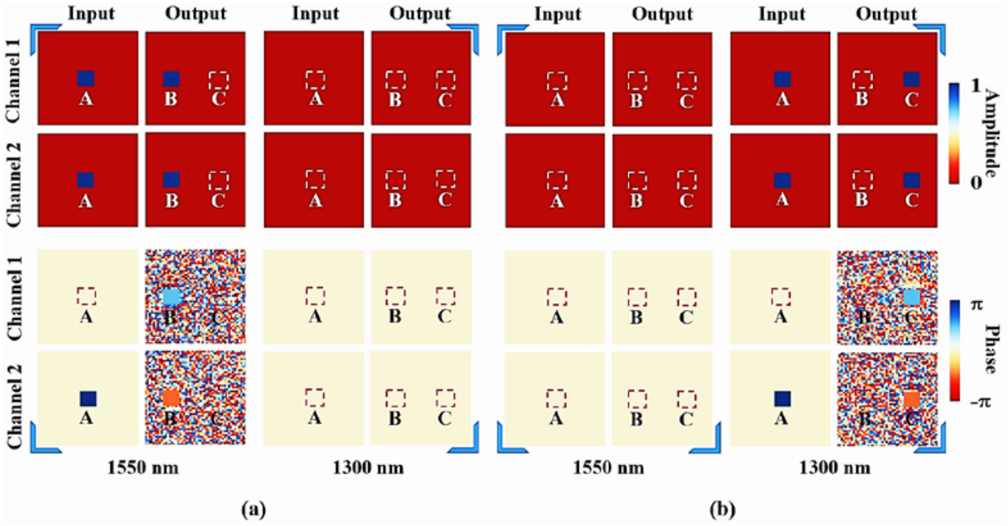

Fig. S7 The training results of the two-input wavelength router under Y-linearly polarized light. The images demonstrate the amplitude and phase distribution of the input and output electric fields in the polarization and wavelength channels. Specifically, it depicts the scenario when the input light has a wavelength of 1550 nm (a), or 1300 nm (b). The final electric field is output at ports B (a), or C (b), respectively

Supplementary Note 4: Three-input wavelength router under Y-linearly polarized light

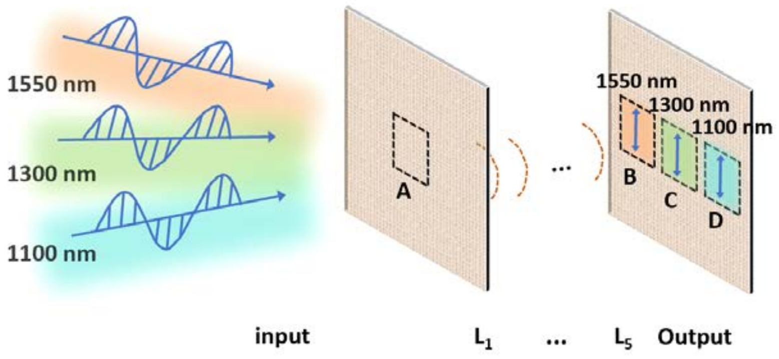

Fig. S8 Schematic diagram of three-input wavelength router under Y-linearly polarized light. A represents the input port of the input layer, while B, C, and D represent the three output ports located at the output layer

Under Y-linearly polarized light, the three-input wavelength router is depicted in Fig. S8. Port A can input light with wavelengths of 1550 nm, 1300 nm, or 1100 nm. When light with wavelengths of 1550 nm, 1300 nm, or 1100 nm is input at port A, it undergoes propagation processing by the diffractive layers and is respectively output at ports B, C, or D.

As illustrated in Fig. S9(a), when Y-linearly polarized light with a wavelength of 1550 nm is input, there is no electric field data in the 1300 nm and 1100 nm channels. In the 1550 nm channel, the electric field amplitudes in Channel 1 and Channel 2 at input port A are both 1, with a phase of 0 in Channel 1 and a phase of π in Channel 2, the input electric field is Y-linearly polarized light with a wavelength of 1550 nm. After propagating through the diffractive layers, the electric field amplitudes in Channel 1 and Channel 2 at output port B are close to 1. The phase in channel 1 is π/2, while the phase in channel 2 is -π/2, resulting in Y-linearly polarized light. In Fig. S9(b), when Y-linearly polarized light with a wavelength of 1300 nm is input, only the 1300 nm channel has electric field data. After propagation through the diffractive layers, the electric field amplitudes in Channel 1 and Channel 2 at output port C are both close to 1. The

phase in channel 1 is $\pi/2$, while the phase in channel 2 is $-\pi/2$, resulting in Y-linearly polarized light. Similarly, in Fig. S9(c), when Y-linearly polarized light with a wavelength of 1100 nm is input, only the 1100 nm channel has electric field data. After propagating through the diffractive layers, the electric field amplitudes in Channel 1 and Channel 2 at output port D are both close to 1. The phase in channel 1 is $\pi/2$, while the phase in channel 2 is $-\pi/2$, resulting in Y-linearly polarized light. The insertion loss of the trained wavelength router is 0.014 dB, and the extinction ratio reaches 20.16 dB, which closely resembles its performance under X-linearly polarized light.

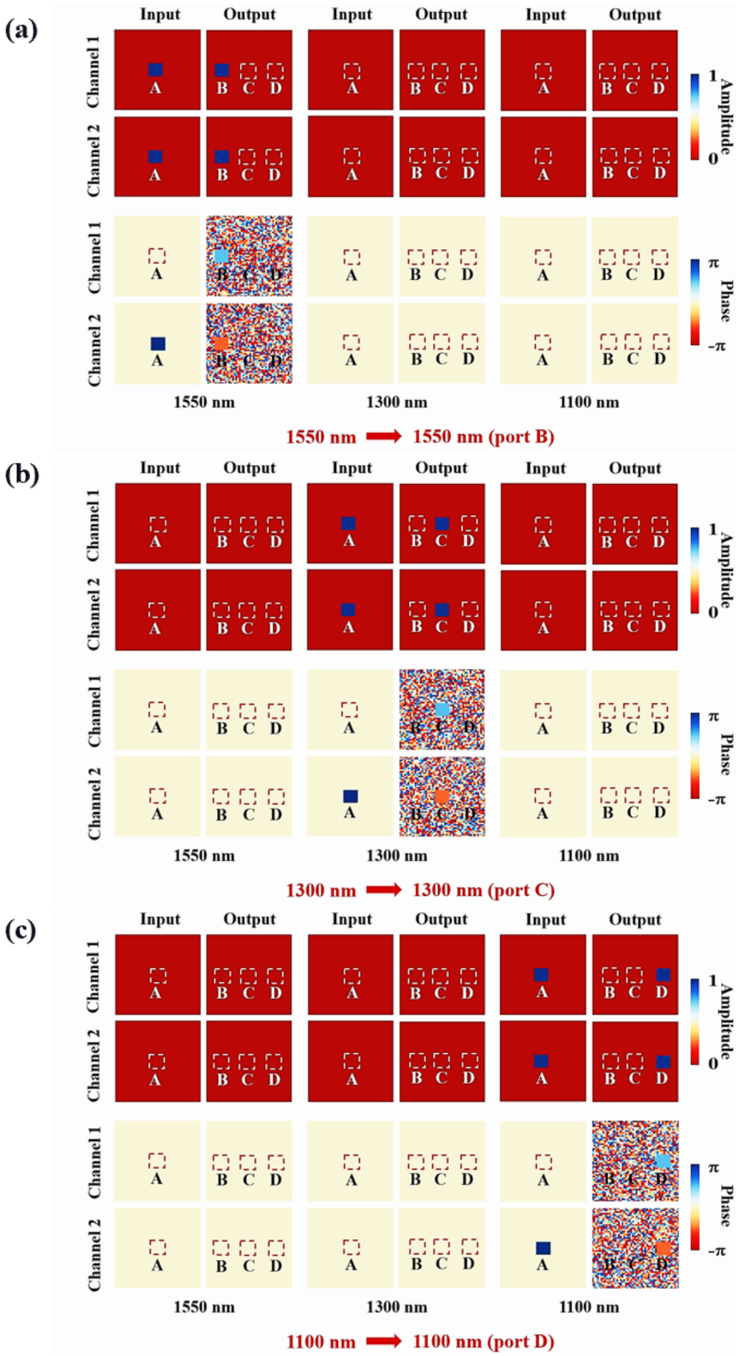

Fig. S9 The training results of the three-input wavelength router under Y-linearly polarized light. The images demonstrate the amplitude and phase distribution of the input and output electric fields in the polarization and wavelength channels. Specifically, it depicts the scenario when the input light has a wavelength of 1550 nm (a), 1300 nm (b), or 1100 nm (c). The final electric field is output at ports B (a), C (b), or D (c), respectively

Supplementary Note 5: Four-input polarization-wavelength composite router

In the four-input polarization-wavelength composite router, the four input electric fields comprise X-linearly polarized light with wavelengths of 1550 nm and 1300 nm, as well as Y-linearly polarized light with wavelengths of 1550 nm and 1300 nm.

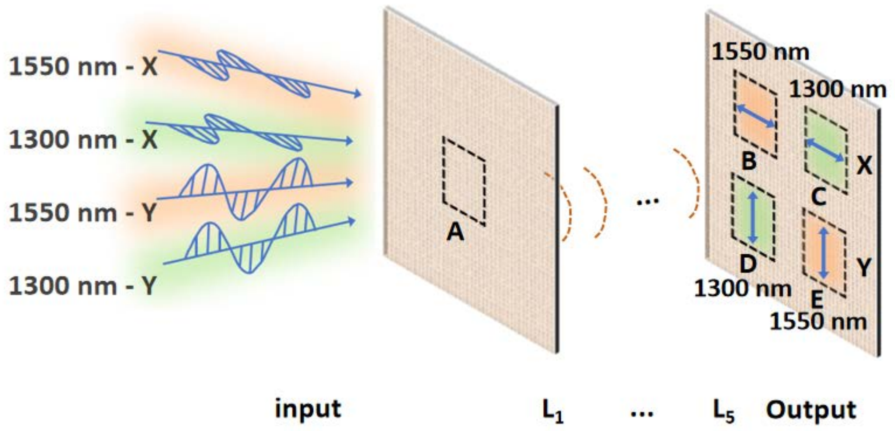

Fig. S10 Schematic diagram of four-input polarization-wavelength composite router. A represents the input port of the input layer, while B, C, D, and E represent the four output ports located at the output layer

Fig. S10 illustrates the schematic diagram of the four-input polarization-wavelength composite router, with port A designated as the input port and ports B, C, D, and E as the output ports. Port A can input four types of electric fields. When X-linearly polarized light with wavelengths of 1550 nm, or 1300 nm is input at port A, it is modulated by the diffractive layer and output at ports B, or C, respectively. When Y-linearly polarized light with wavelengths of 1300 nm, or 1550 nm is input at port A, it is modulated by the diffractive layer and output at ports D, or E, respectively.

When X-linearly polarized light with a wavelength of 1550 nm is input at port A, as shown in Fig. S11(a), there is no electric field data in the 1300 nm channel. In the 1550 nm channel, the electric field amplitude for both channels 1 and 2

at input port A is 1, with phases of 0. Therefore, the input electric field is X-linearly polarized light with a wavelength of 1550 nm. After propagating through the diffractive layers, the electric field is ultimately output at port B, with both channel 1 and channel 2 having electric field amplitudes close to 1 and phases around π/2, resulting in X-linearly polarized light. Fig. S11(b) illustrates the electric field distribution in each channel when the input is X-linearly polarized light at 1300 nm, with the electric field ultimately output at port C as X-linearly polarized light. When Y-linearly polarized light with a wavelength of 1300 nm is input at port A, as illustrated in Fig. S12(a), only the 1300 nm channel has electric field data. The electric field amplitude at input port A for both channels 1 and 2 is 1, with a phase of 0 in channel 1 and a phase of π in channel 2. After propagating through the diffractive layers, the electric field with a wavelength of 1300 nm is ultimately output at port D, with both channel 1 and channel 2 having electric field amplitudes close to 1. The phase in channel 1 is π/2, while the phase in channel 2 is -π/2, resulting in Y-linearly polarized light. When Y-linearly polarized light with a wavelength of 1550 nm is input at port A, as depicted in Fig. S12(b), only the 1550 nm channel has electric field data. After propagating through the diffractive layers, the electric field is output at port E, with both channel 1 and channel 2 having electric field amplitudes close to 1. The phase in channel 1 is π/2, while the phase in channel 2 is -π/2, resulting in Y-linearly polarized light.

After training, the four-input polarization-wavelength composite router exhibits an insertion loss of 1.34 dB, and an extinction ratio of 10.15 dB. Its performance is comparable to that of the six-input polarization-wavelength composite router.

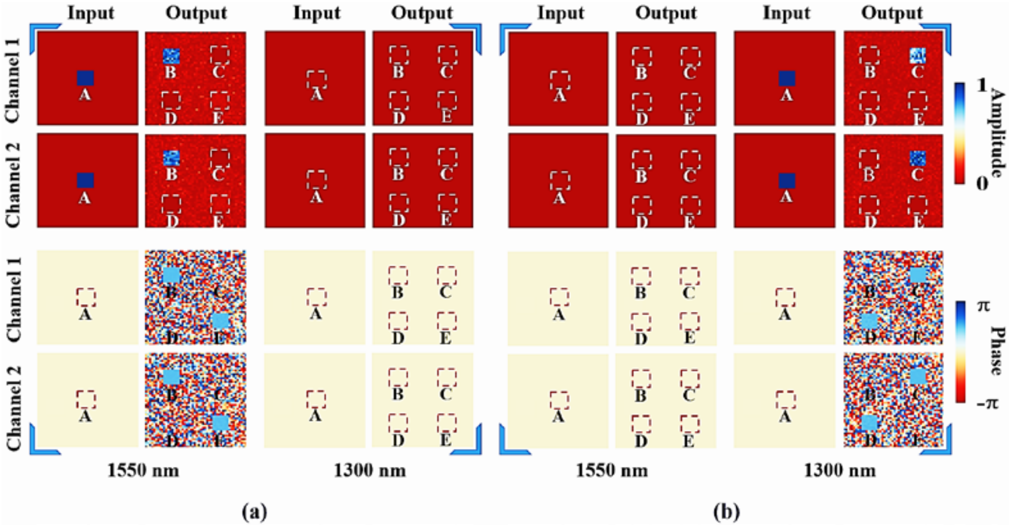

Fig. S11 The training results of the four-input polarization-wavelength composite router. The images demonstrate the amplitude and phase distribution of the input and output electric fields in the polarization and wavelength channels. Specifically, it depicts the scenario when the input light is X-linearly polarized with a wavelength of 1550 nm (a), or when it is X-linearly polarized with a wavelength of 1300 nm (b). The final electric field is output at ports B (a), or C (b), respectively

During each computation of the router, only the signal is output at the target port, while the output at other non-target ports is considered noise. We calculate the crosstalk (CT) by evaluating the ratio of the optical intensity at non-target output ports to the total input optical intensity:

$$CT = 10 \log_{10}\left(\frac{I_{\text{noise}}}{I_{\text{in}}}\right), \tag{7}$$

where I_{noise} represents the optical intensity of the non-target output ports. And I_{in} is the total input optical intensity, and in the calculation process, the optical intensity of each input type is the same.

The crosstalk values for the polarization router, two types of two-input wavelength routers, two types of three-input wavelength routers, four-input and six-input polarization-wavelength composite routers are as follows: -8.65 dB, -

25.40 dB, -22.52 dB, -18.98 dB, -20.18 dB, -11.74 dB and -13.26 dB, respectively.

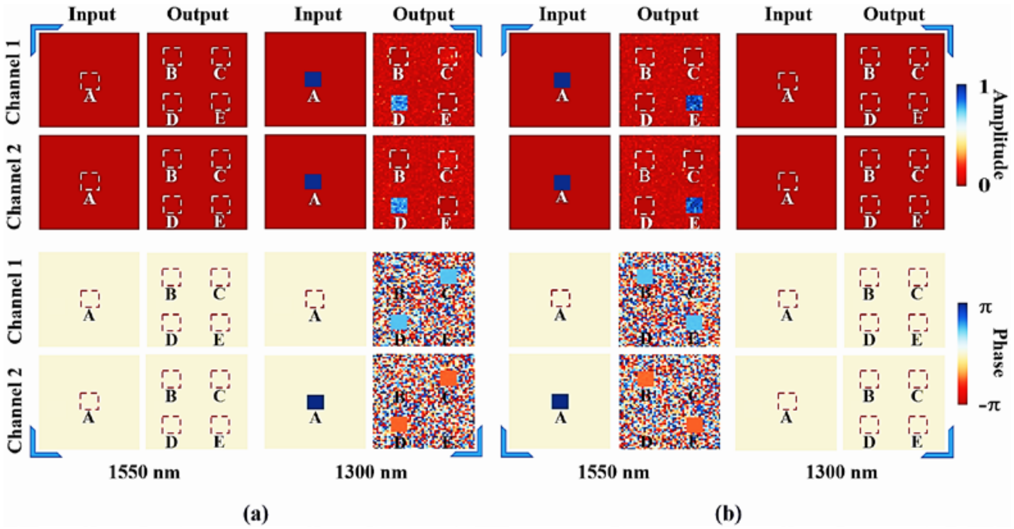

Fig. S12 The training results of the four-input polarization-wavelength composite router. The images demonstrate the amplitude and phase distribution of the input and output electric fields in the polarization and wavelength channels. Specifically, it depicts the scenario when the input light is Y-linearly polarized with a wavelength of 1300 nm (a), or when it is Y-linearly polarized with a wavelength of 1550 nm (b). The final electric field is output at ports D (a), or E (b), respectively
